# The continuous actuation of liquid metal with a 3D-printed electrowetting device

**DOI:** 10.1007/s44258-025-00052-8

**Published:** 2025-04-01

**Authors:** Samannoy Ghosh, Rajan Neupane, Dwipak Prasad Sahu, Jian Teng, Yong Lin Kong

**Affiliations:** Department of Mechanical Engineering, Rice University, Houston, TX 77005 USA

**Keywords:** Electrowetting, Liquid metal actuation, Cargo transport, 3D printing

## Abstract

The ability of liquid metals (LMs) to recover from repeated stretching and deformation is a particularly attractive attribute for soft bioelectronics. In addition to their high electrical and thermal conductivity, LMs can be actuated, potentially enabling highly durable electro-mechanical and microfluidics systems for applications such as cooling, drug delivery, or reconfigurable electronics. In particular, continuous electrowetting (CEW) phenomena can actuate liquid metal at relatively low voltage and affordable power requirements for wearable systems (~ < 10 V, ~ 10 – 100 µW) by inducing a surface tension gradient across the LM. However, sustaining LM actuation remains challenging due to factors such as electrolyte depletion, polarity changes in multi-electrode systems, and limitations related to LM composition. Here, we demonstrate LM actuation in a circular conduit for prolonged durations of at least nine hours. We enabled sustained actuation by sequentially applying short, direct current (DC) pulses through a multi-electrode system based on the dynamics of LM actuation. As a proof of concept, we also demonstrated the ability of LM to transport electrically conducting, non-conducting, and magnetic materials within a microchannel and show the liquid metal actuation system can be potentially miniaturized to the size of a wearable device. We envision that with further miniaturization of the device architectures, our CEW platform can enable future integration of low-voltage electro-mechanical systems into a broad range of wearable form factors.

**Graphical Abstract:**

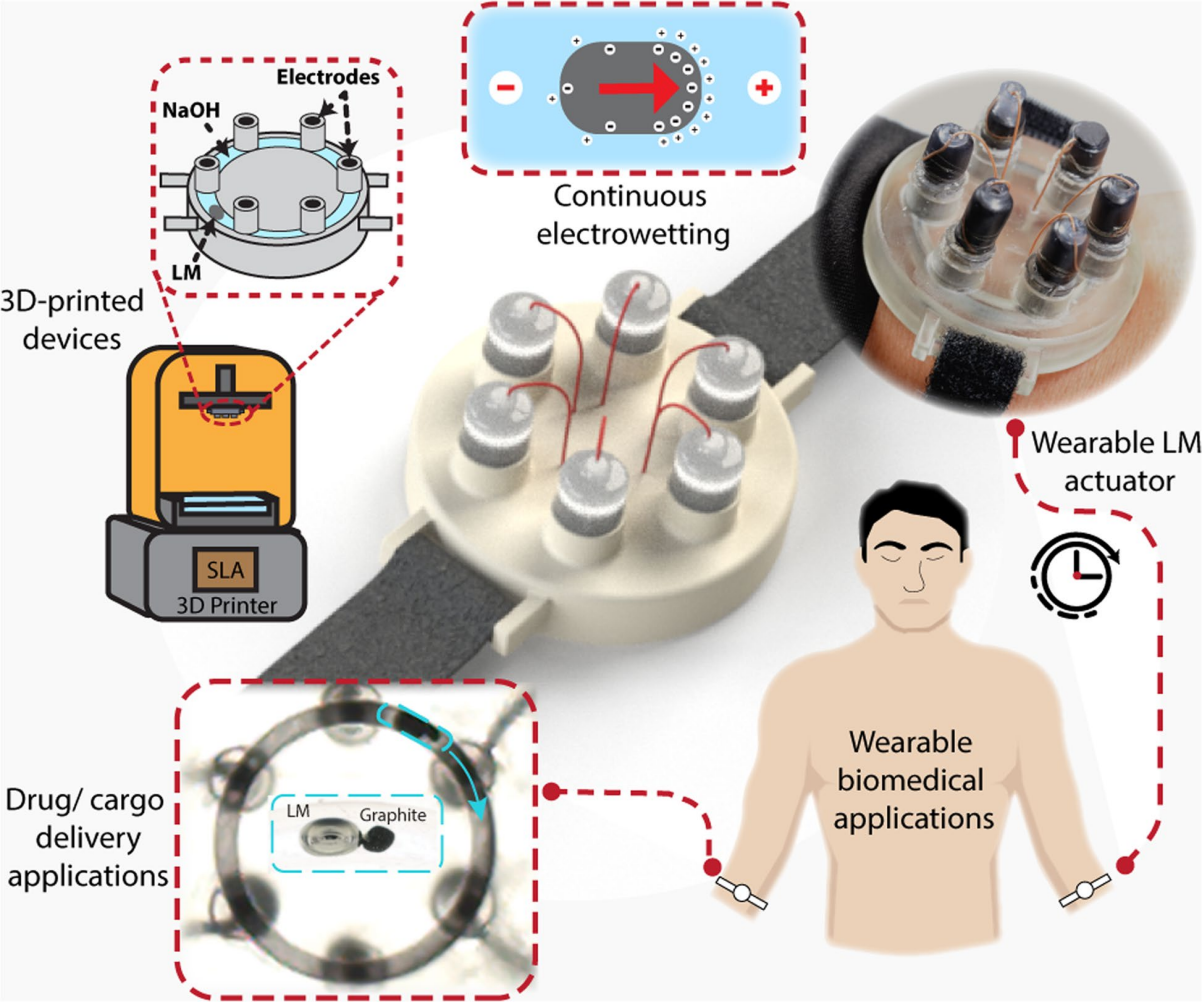

**Supplementary Information:**

The online version contains supplementary material available at 10.1007/s44258-025-00052-8.

## Highlights

- We achieved prolonged LM actuation in a circular conduit of at least nine hours.
- We enabled sustained actuation by sequentially applying short, direct current (DC) pulses through a multi-electrode system based on the dynamics of LM actuation.
- The platform can also transport electrically conductive, insulating, and magnetic composite cargos in a channel.

**Supplementary Information:**

The online version contains supplementary material available at 10.1007/s44258-025-00052-8.

## Introduction

Advances in materials innovation can enable the creation of wearable and epidermal devices with increasingly sophisticated bioelectronic capabilities [1–4] in health monitoring [5–9], physical activity tracking [10, 11], and drug delivery [12, 13], even in soft, flexible, and stretchable constructs [14, 15]. Liquid metals (LMs) are a unique class of metals that exist as a liquid at room temperature, which allows them to simultaneously maintain high electrical conductivity and mechanical flexibility [16], enabling the creation of exceptionally robust textile electronics [17] and stretchable circuits [18].

In addition to being used as flexible electrical or thermal conductors, LMs can also be actuated via electrocapillarity, continuous electrowetting (CEW), electrowetting on dielectric (EWOD), reverse electrowetting (RWOD) effects—enabling the creation of electro-mechanical systems such as micropumps [19–22]. In particular, CEW can actuate LM without requiring high voltage or power, which are desirable attributes for wearable electronics [23]. Specifically, CEW is generated when applied electric potential induces a surface tension gradient across the LM [22–25] with a potential lower than 10 V [26].

Prior work has leveraged CEW as micropumps [19, 20], microvalves [27, 28], sensing elements [29], cargo transport [30, 31], radio-frequency (RF) applications [32, 33], and micromotors [34]. For example, LM enables the manipulation of micro and nanoliters of fluid inside a microchannel for biomedical applications [19, 35] Yun et al. reported the actuation of mercury using square wave voltage to pump water in a micropump [19]. In another example, Gough et al. utilized CEW to manipulate the LM slug as a tunable RF device [33].

Zhang et al. explored the use of an external electric or magnetic field to actuate LM droplets by leveraging the electrochemical reactions between LM and aluminum, enabling the transport of hydrogel samples inside a millimeter-sized channel [36]. However, the sustained propulsion of the LM was hindered by the fast consumption of the aluminum foil. In another example, Liu et al. utilized an external magnetic field to steer LMs in both solid surfaces and liquid environments [31]. By encapsulating micro-sized steel beads in the LM droplet, they achieved magnetic control of the droplet motion, though a thermal heating process was required to guide and release gelation-based cargo.

Indeed, previous work has underscored the potential of CEW of LMs in realizing a broad range of powerful potential applications, from microscale transport to tunable RF devices. However, sustaining LMs with the current approach remains challenging due to surface oxidation, electrode polarization, electrolysis of the electrolyte, and changes in the amounts of reactants [36, 37]. For example, surface oxidation under the effect of electric fields deteriorates the performance of the LM by altering the surface tension. Additionally, the loss of eutectic ability of the gallium-based LM due to the dissolution of gallium in the electrolyte leads to a change in their melting point and structural integrity, which further impacts the sustained actuation of LM [20, 38, 39].

Here, we investigate strategies for prolonged CEW actuation of LMs in the conduit with potential future applications in wearable devices. We designed circular electrowetting devices that are digitally manufactured using a stereolithography 3D printer and demonstrated the ability to sustain unprecedented long continuous operation (at least 9 h), a significant improvement over prior CEW-based actuation that can only sustain the actuation for less than one hour [22]. We also demonstrated the ability of the actuated liquid metal to generate forces capable of transferring cargo inside the conduit.

Specifically, we enabled the sustained actuation by sequentially applying short, direct current (DC) pulses through a multi-electrode system, which is optimized based on our investigation of the dynamics of LM actuation. For example, we studied the influence of geometrical and volumetric parameters on the motion of the LM by regulating the commutation period. We determined the minimum potential difference required for the LM to seamlessly complete continuous revolution inside the channel for various sizes of LM. We also explored electrolyte concentration’s influence on LM’s movement and actuation over extended hours. Finally, as a demonstration of the magnitude of actuation forces and potential applications, we show the ability of LM to transport electrically conducting, non-conducting, and magnetic materials within a microchannel and demonstrate that such a complex continuous actuation system can be potentially miniaturized to the size of a wearable device.

## Experimental setup

Additive manufacturing enables the scalable fabrication of devices, including microfluidic devices, that can be tailored for specific applications [40–42]. Here, we leverage stereolithography (SLA) capability to achieve rapid design iteration, prototyping, and optimization [43] (Form 3B, Formlabs, USA) and ultimately create transparent microfluidic circular channel devices with clear resin. Specifically, 3D models were prepared in SolidWorks (Dassault Systems Inc.) and converted to stereolithography (STL) files. The STL files were imported to a 3D slicing software (Preform), and the highest resolution for layer height (25 μm) setting was used to print the devices.

After printing, the devices were washed in an IPA bath and cured in the UV chamber for 30 min at 60 °C. Furthermore, we dispensed approximately 20 mL of the clear resin on the backside of the devices and coated the device with the resin using a fine brush. The devices were then cured in the UV chamber for 200 more minutes to allow the clear resin to seep into the remaining pores of the 3D printed devices, which we found to have improved the transparency for image acquisition. The additional resin fills the surface voids, thereby reducing light scattering.

Graphite electrodes of various diameters (2, 3, and 6 mm) were purchased from McMaster Carr and used in the 3D-printed post slots in the device. The different concentrations of electrolyte solutions were prepared by dissolving sodium hydroxide flakes (Sigma Aldrich, USA) in deionized water. Eutectic gallium indium (eGaIn) was purchased from Sigma Aldrich and loaded in 250 µL syringes (Hamilton Inc., USA) to pump into the fabricated devices using a micropump (Harvard Apparatus, USA).

**Fig. 1:**
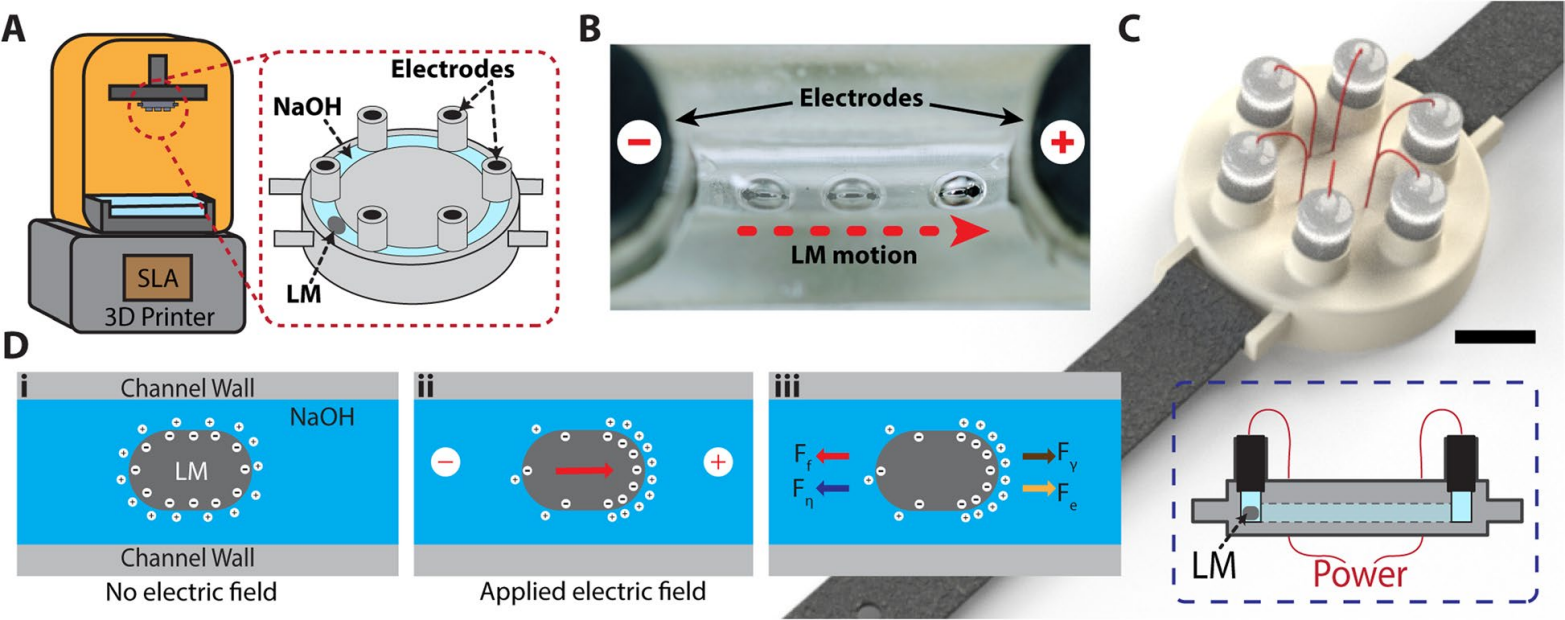
A 3D-printed device demonstrating liquid metal (LM) actuation via continuous electro-wetting (CEW) in a circular conduit, with potential applications in wearable electronics **(A)** A schematic illustration of the 3D-printed device demonstrating CEW in a circular channel integrated with a series of electrodes. **B** The top view of a microfluidic channel with electrodes actuating the liquid metal droplet towards the positive electrode upon excitation. **C** 3D rendering of the wearable prototype with CEW LM actuation in the form factor of a watch. The scale bar is 10 mm. The inset shows the cross-sectional schematic of the prototype. **D** (i) The uniform electric charge distribution along the surface of the liquid metal in the absence of an electric field. (ii) The induced surface tension gradient due to charge reconfiguration when an electric field is applied. (iii) The force balance on the liquid metal inside a 3D-printed microchannel when an electric potential is applied across the two ends of the channel. The motion of liquid metal is modulated by the surface tension force F_γ_, electro-osmotic force F_e_, friction force F_f_, and viscous force F_η_

The experimental device consisted of a resin-printed circular channel of various sizes featuring six electrode posts equipped with graphite electrodes. The channel was filled with NaOH electrolyte solution, followed by a careful introduction of a 10 μL LM droplet. Figure 1A shows the schematic of the 3D-printed device with the circular channel filled with NaOH and the liquid metal droplet. We used the concept of an H-bridge circuit to apply direct current (DC) to the electrodes. The purpose of the H-bridge is to enable bidirectional current control and provide power isolation from control signals.

A microcontroller (Arduino Uno) was programmed to trigger the signal for a specified interval using a computer. A power supply was connected to the H-bridge circuit, and the output of the circuit was connected to the electrodes. The magnetic polymer composite for cargo transport was prepared by casting addition-cure silicon (Dragon Skin 10 Medium, Smooth-On) in a 3D-printed mold [44]. Equal parts of A: B components were mixed in a planetary centrifugal mixer (AR-100, Thinky) for 60 s at 2000 rpm. After injecting the mixture into the mold, the parts were cured overnight and cut into desired sizes using a fine blade. A high-speed camera (IDT NX4-S3) was used to capture the motion of the liquid metal inside the microchannel, and image processing was used to estimate the velocity of the liquid metal inside the microchannel. Another high-speed camera (Photron FastCam mini UX50) and Dinolite digital microscope were used for recording videos of the cargo delivery experiments.

## Design principle and theoretical modeling

Previous studies have discussed the theoretical understanding of LM actuation and control in millimeter-scale channels [37, 45, 46]. Figure 1B highlights the experimental demonstration of the LM actuation in a microfluidic channel, showing its movement from the negative to the positive electrode upon application of an electric field. The motion of the LM inside the channel is dictated by interfacial surface tension, viscous drag from the electrolyte, frictional interaction with the substrate, and electroosmosis force [46, 47]. Figure 1C shows the conceptual 3D rendering of a wearable prototype in the form factor of a watch. The inset shows the cross-sectional view and how the LM is contained in the channel with the graphite electrodes touching the NaOH.

Here, we also highlight the contribution of each of the forces to the motion of the LM inside the channel and show its relation with various electrical and geometrical parameters. Initially, in the absence of the external electrical potential, the electric double layer (EDL) is uniformly distributed along the surface of the LM [48, 49]. The cohesive force between the charged particles at the surface of the LM is influenced by factors such as electrolyte concentration, the type of the LM used, and the magnitude of applied electrical potential across the electrode. Figure 1D(i) shows the distribution of the ions on the surface of the LM when immersed in an electrolyte solution.

CEW is the result of the electrocapillary effect, which describes the change in interfacial tension at the liquid–metal interface due to an applied electric potential. Assuming the charge in the EDL is uniformly distributed in the LM. To model the capillary pressure difference of the LM immersed in the electrolyte solution, we use the Young–Laplace equation,

1 $$\Delta P=\gamma \nabla \bullet {\varvec{n}},$$

where $\Delta P$ is the pressure difference between the ends of the LM and electrolyte, $\gamma$ is the surface tension, and ${\varvec{n}}$ is the unit outward normal to the surface. When the LM is deformed into an ellipsoid shape, Eq. 1 can be expressed as

2 $$\Delta P=\gamma \left(\frac{1}{{\text{R}}_{1}}+\frac{1}{{\text{R}}_{2}}\right),$$

where ${\text{R}}_{1}$ and ${\text{R}}_{2}$ are the principal radii of curvature. In our experimental setup, the LM is not confined by the microfluidic channel and maintains a spherical-like shape. For simplicity, we assume the LM is spherical and has a uniform radius similar to previous studies [20, 45, 49]. Under these conditions, the Young–Laplace equation [20, 45] is simplified to

3 $$\Delta P=\frac{2\gamma }{\text{r}},$$

where, $\text{r}$ is the radius of the LM. The radii of curvature at both hemispheres are assumed to be equal in a uniformly sized microchannel.

Under an external electrical field, the surface tension at the two ends of the LM differs, leading to a pressure difference that can be expressed as

4 $$\Delta P={P}_{L}-{P}_{R}=\frac{2\left({\gamma }_{L}-{\gamma }_{R}\right)}{r}=\frac{2\Delta \gamma }{r},$$

where ${P}_{L}$ and ${P}_{R}$ denote the pressure differences between electrolytes and LM’s left and right ends, respectively; ${\gamma }_{L}$ and ${\gamma }_{R}$ are the surface tension of the LM’s left and right ends, respectively. Equation 4 can be further expanded using Lippmann’s equation [50], and the surface tension difference can be expressed in terms of potential difference at the two ends of LM,

5 $$\Delta \gamma =\frac{c\left({V}_{L}^{2}-{V}_{R}^{2}\right)}{2}.$$

Here, ${V}_{L}$ and ${V}_{R}$ are the potential differences across the EDL at the left and right ends of the LM, respectively, and $c$ is the initial capacitance per unit area of the EDL in the absence of an external electrical field. Equation 5 shows that the interfacial surface tension difference depends on the potential difference across the LM. Since the initial capacitance of the EDL is a constant for a given liquid metal and electrolyte combination, a larger potential difference results in a greater surface tension difference. Also, the initial charge density is a function of the electrolyte concentration, the type of LM, and the temperature of the electrolyte solution [51].

Using Eq. 5 and considering an applied potential difference across the two ends of the LM, previous studies [20, 45] have shown that the pressure difference between two ends of the LM can be expressed as,

6 $$\Delta P=\frac{2\Delta \gamma }{r}=\frac{4{q}_{0}{A}_{current}}{\left({A}_{current}-\frac{2\pi {r}^{2}}{3}\right)}\frac{{V}_{electrode}}{L},$$

where ${q}_{0}$ is the initial charge per unit area in EDL, ${A}_{current}$ is the cross-sectional area of the current path, ${V}_{electrode}$ is the potential applied across the electrodes, and $L$ is the length of the total current path. By integrating over the surface area of the LM, the surface tension force can be calculated as,

7 $${F}_{\gamma }=\pi {r}^{2}\Delta P=\frac{4\pi {r}^{2}{q}_{0}{A}_{current}{V}_{electrode}}{\left({A}_{current}-\frac{2}{3}\pi {r}^{2}\right)L}.$$

The acceleration of the LM inside the channel due to surface tension force can then be calculated using$a={F}_{\gamma }/m$, where $m$ is the mass of the LM. Equation 7 highlights the dependence of the surface tension force on parameters such as applied voltage, LM size, and the current path length. Notably, the surface tension force is inversely proportional to$L$, meaning that shorter current paths result in higher forces. Even though Eq. 7 is derived under an equilibrium state that assumes enough replenishment, parameters (i.e. ${q}_{0}$,$r$, and${V}_{electrode}$) in Eq. 7 can exhibit time dependence due to factors such as electrolyte depletion or oxidation if the electrolyte is not replenished. In our study, we did not observe changes due to electrolytes within the time scale of our research and we assumed that parameters in Eq. 7 are constant.

Furthermore, the motion of the LM is opposed by retardation forces such as friction force and viscous drag, as illustrated in Fig. 1D. When moving in a channel filled with a viscous fluid, the LM experiences a drag force opposing its motion, given by Stokes’ drag:

8 $${F}_{\eta }=6\pi \eta rv,$$

where $\eta$ is the viscosity of the electrolyte and $v$ is the velocity of LM. Additionally, the moving LM experiences a friction force between the surface of the LM and the channel surface, which can be expressed as,

9 $${F}_{f}={\rho }_{df}{V}_{LM}g\beta$$

where ${\rho }_{df}$ is the density difference between the LM and the electrolyte, ${V}_{LM}$ is the LM volume, $g$ is the gravitational acceleration, and $\beta$ is the coefficient of friction between the LM and the surface.

## Results and discussion

As a proof-of-concept, we conducted a series of experiments to investigate the behavior of a liquid metal (LM) droplet in an open-top circular conduit actuated by a six-electrode system under varying conditions. We examined the minimum operational voltage required to demonstrate continuous electrowetting for LM droplets ranging from 5.0 µL to 30.0 µL and complete continuous revolutions in the channel. Additionally, we measured the average and instantaneous velocity of the LM at different switching frequencies of the applied potential. The influence of electrode dimensions on LM velocity was also investigated using various electrode sizes. Long-term actuation tests lasting up to nine hours were performed to evaluate the LM velocity profile and the effects of periodic electrolyte replenishment within the channel. Finally, we demonstrated the system’s capability to transport electrically conductive, insulating, and magnetic composite cargos while also showcasing its functionality in a closed-top, wearable form factor.

In this study, we utilized electric potential to actuate LMs by leveraging the continuous electrowetting phenomenon to dynamically control surface tension. When the LM is immersed in an electrolyte solution such as sodium hydroxide (NaOH), it undergoes a chemical reaction with the electrolyte, producing gallates [Ga(OH)] as per the equation [20]:

10 $$\text{2Ga + 2NaOH + 6H}_{2}\text{O} \rightarrow 2Na [Ga (OH)_{4}]^{-1}\text{ + 3H}_{2}$$

This reaction results in a distribution of the anions on the surface of liquid metal, rendering the surface negatively charged as shown earlier in Fig. 1D (i). While hydrogen gas will also be produced, it is not significant [52] below 9 V when NaOH is used as an electrolyte and does not contribute to LM propulsion via bubble thrust force [36, 37, 53, 54]. Previous works with aluminum, zinc, nickel, or tungsten as fueling agents reported the formation of bubbles and bubble thrust as LM propulsion mechanisms [55]. However, there may be some potential buildup of gas bubbles over extended durations. These can be mitigated by introducing venting mechanisms and channels covered with gas-permeable hydrophobic membranes [56] that will allow trapped gas bubbles to diffuse out of the channel while maintaining an enclosed system to prevent electrolyte loss.

It is worth noting that the surface chemistry and ion absorption are highly dependent on the composition of the LM and the electrolyte. For example, when a mercury droplet is immersed in a NaOH solution, cations surround the surface of liquid metal [22] whereas eGaIn forms a layer of anions on its surface. These negative charges on the surface of the LM attract positive ions from the solution, forming a thin, diffuse layer of EDL. The thickness of this EDL depends on the LM composition, electrolyte concentration, solution temperature, and applied electric potential across the electrolyte solution [57]. Moreover, the EDL acts like a charged capacitor due to the accumulation of opposite ions at the LM-electrolyte interface, with the LM maintaining uniform conductivity due to its high intrinsic conductivity [20]. However, when an external potential is applied, the finite conductivity of the electrolyte generates a potential gradient along the surface of LM, causing charges to redistribute. This results in a surface tension gradient, as illustrated in Fig. 1D(ii), which propels the LM towards the region of lower surface tension—a phenomenon interpreted as surface energy minimizations by wetting the lower surface tension region more than the higher one.

**Fig. 2:**
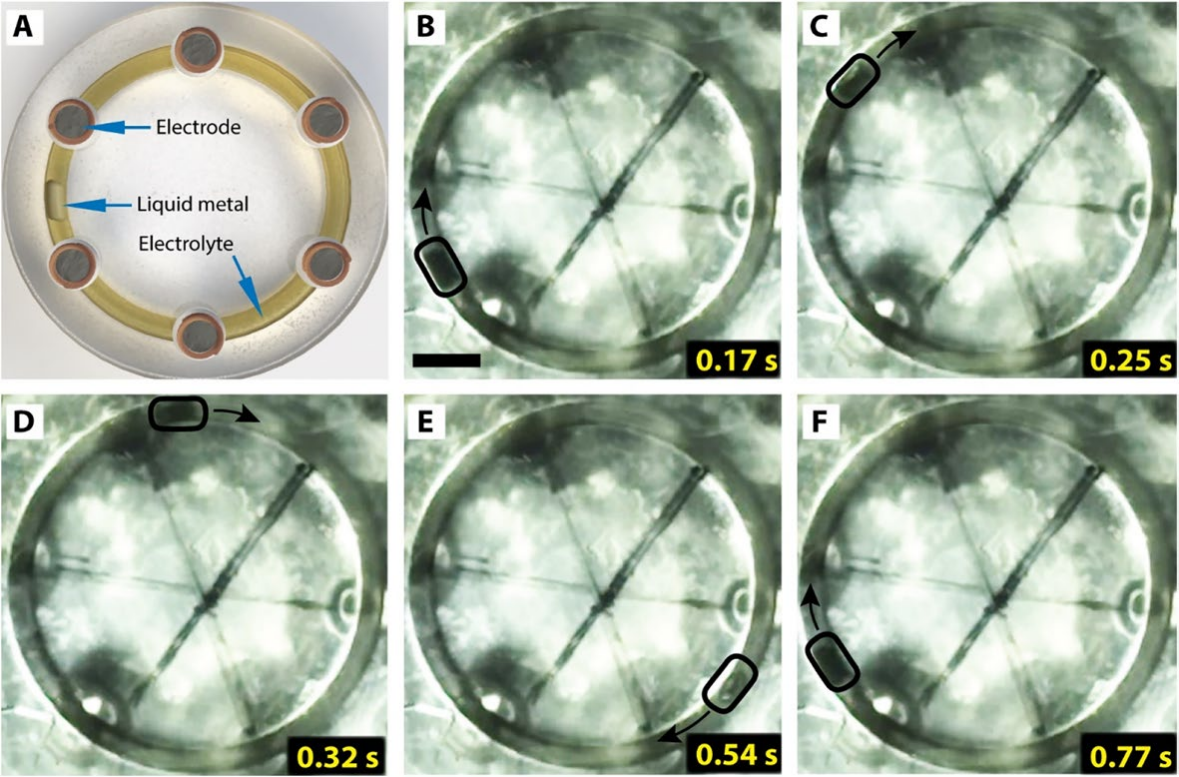
Real-time actuation of the liquid metal (LM) immersed in NaOH electrolyte, under applied electric field, as visualized using high-speed camera. **A** Schematic of a circular microchannel with a liquid metal under six graphite electrodes. Figures (**B**-**F**) are images of the LM at different timestamps when the LM makes a complete revolution in a clockwise direction. The channel is 2.5 mm wide, 6 mm deep and contains 2.5 mm long electrodes in a 0.4 M NaOH electrolyte. The scale bar is 5 mm

To address our research objective, we 3D-printed circular conduits with a rectangular cross-section (3 mm wide × 6 mm deep) having varying channel diameters (26 mm, 28 mm, 30 mm, and 32 mm). We note that the channel diameter here refers to the diameter of the circular channel of the microfluidic device, not the cross-section of the channel. A 10 μL LM droplet was then injected into the channel, and its motion was studied under a sequentially applied electric field. Figure 2A shows the schematic of our device featuring six evenly spaced electrode posts with a diameter of 3.2 mm. During the LM actuation in these channels, it was observed that the LM achieved continuous full-cycle displacement in the 26 mm diameter channel under 8.7 V DC field with a 0.4 mol L⁻^1^ NaOH solution. In contrast, LM exhibited intermittent stops in larger diameter channels and thus failed to maintain continuous cyclic motion. Specifically, the LM in larger channels tended to settle between two adjacent electrodes, initiating oscillatory motion rather than completing the full cycle. This behavior likely stems from an insufficient voltage drop (∆φ) across the LM’s two ends, preventing the formation of sufficient surface tension gradient necessary to drive the LM between the electrodes.

From the tests performed with the different channel diameters, we determined the 26 mm channel diameter to be the optimal device size for our subsequent CEW characterization tests. Tests performed with larger diameters failed to consistently actuate the LM, resulting in an oscillatory motion. Time-series images from the video (supporting information Online Resource 1) capture the motion of LM within the channel. The voltage was applied to the electrodes sequentially, with each electrode powered on for about 150 ms. The electrodes were continuously polarized in a single direction, maintaining a commutation sequence that allowed ceaseless motion of the LM. The LM consistently traveled from the negative to the positive electrode, aligning with previous studies [49, 58]. Figure 2 (B-F) highlights the LM completing a full cycle around the channel in less than a second, demonstrating the precise control of electrode excitation and modulation of surface tension.

**Fig. 3:**
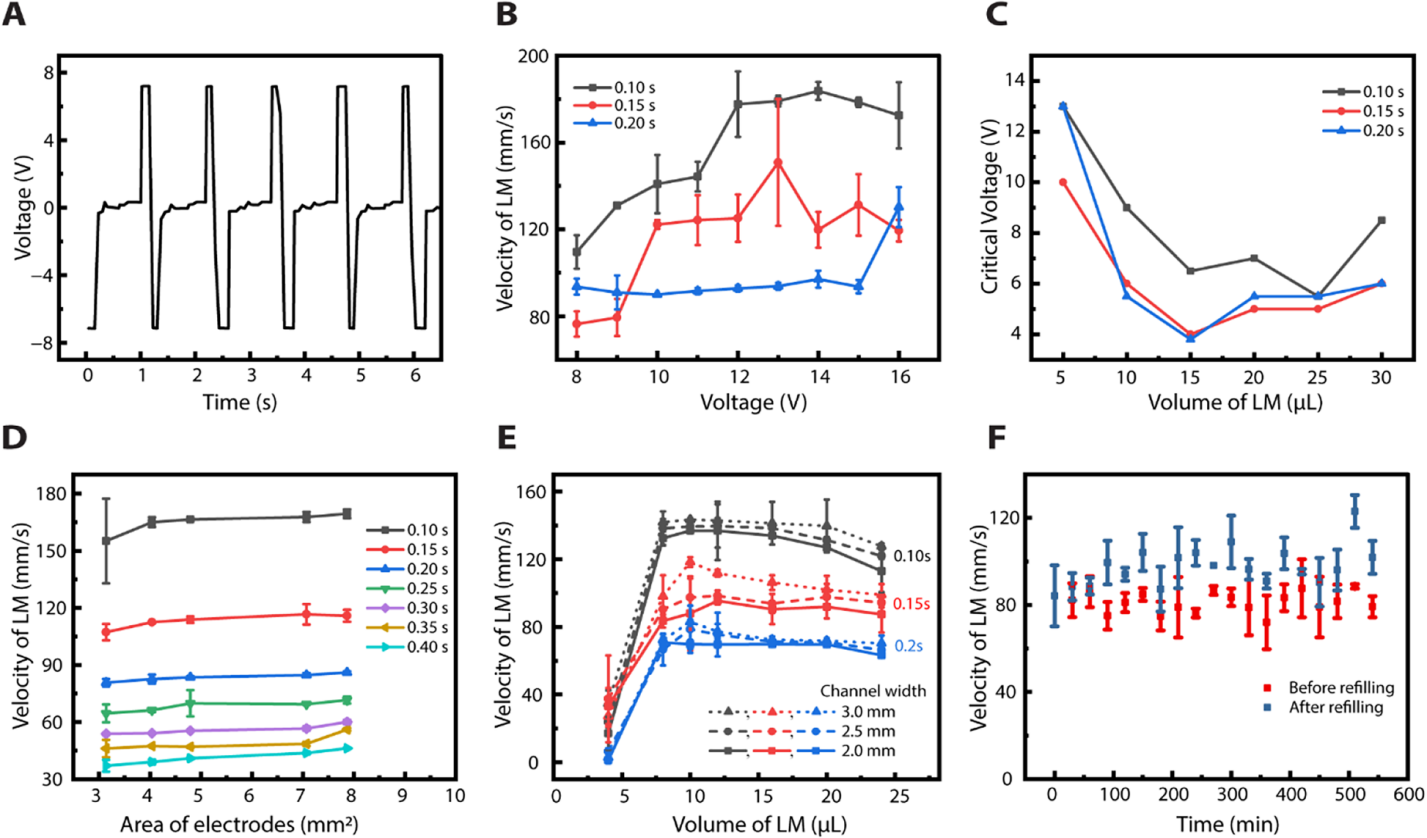
Characterization of the electrical pulses and velocity of LM under different parameters. **A** The electrical potential difference between two adjacent electrodes when 8.7 V is supplied from the power source (**B**) Velocity of the LM at different voltages (**C**) Minimum voltage required for LM of different volumes (**D**) Velocity of LM in channels with different cross sectional area of electrodes for different commutation periods (**E**) Velocity of liquid metal when different volumes of LM are injected into the microchannel of varying channel width under varying commutation periods (**F**) The velocity of LM before (red) and after (blue) refilling electrolyte when a continuous potential difference is applied to the device. **B**, **C** and **E** are performed at different commutation periods of 0.1 s, 0.15 s and 0.2 s

Figure 3A depicts the waveform of the input voltage applied across the six-electrode system, measured between two adjacent electrodes. The voltage alternates between + 7 V and −7 V, corresponding to the sequential activation of the electrodes. As the subsequent electrode is excited, the previous one is grounded. The voltage drops to near zero between these transitions as the other four electrodes are momentarily active, highlighting the crests in the voltage plot. The LM moves consistently toward the positively charged electrode, enabling controlled continuous electrowetting (CEW) actuation.

Previous studies have extensively explored the behavior of electrodes immersed in various electrolytes and ionic conductors [59, 60]. When an electric field is applied, several reactions can occur within the electrolyte, influenced by factors such as electric field strength, chemical diffusion, and thermal diffusion [59]. Also, prior work has discussed the response of metal electrodes when subjected to AC electric field [60–65]. As electric current flows through the electrodes, the potential across two consecutive electrodes is observed to be less than that of the applied potential from the source due to electrode polarization, which involves the accumulation of charge around the electrolyte that forms an electric double layer (EDL) at the electrode–electrolyte interface [66].

When subjected to AC electric field, polarization is manifested as the development of polarization impedance, while DC polarization is characterized by the increase in charge accumulation with the duration of applied current. Essentially, the period of the electrical field application plays a crucial role in the polarization of the electrodes. Equivalent electrical models have been developed to explain physical phenomena at the electrode–electrolyte interface [59, 67]. Franks et al. modeled an equivalent circuit consisting of an interface capacitance, shunted by a charge transfer resistance, in series with the electrolyte resistance [61]. When electrodes are immersed in an electrolyte, an EDL forms around the electrode with a finite strength of electric field. In DC conditions, the polarization builds up in a specific direction, opposing the direction of an applied electric field to facilitate current conduction through the electrolyte. This results in an increased resistance from the EDL, reducing the apparent potential measured across two electrodes. For instance, when a voltage of 8.7 V is supplied, only 7 V is realized across the two consecutive electrodes, as shown in Fig. 3A.

To characterize the LM motion under varying applied voltages, we performed a series of experiments by changing voltages between 8 and 16 V for different commutation periods, as shown in Fig. 3B. We observed that LM velocity increases with voltage up to a threshold limit, for all tested commutation period, beyond which further increase in voltage results in a diminishing return or reduction in velocity. The increased LM velocity can be due to the stronger electric field at higher voltages, resulting in a larger surface tension gradient across the LM droplet that lead to higher acceleration of the LM.

A shorter commutation period resulted in a smoother movement of LM along the circular channel, leading to higher LM velocity. The highest velocity achieved with a 0.1 s commutation period is 183 mm/s at 14 V. The plateau-like behavior at higher voltages for a 0.1 s commutation period may be attributed to the saturation of the electric field strength to further increase the surface tension gradient or limitations in the charge redistribution dynamics on the LM surface. The drop in the LM velocity at 16 V for a shorter commutation period could be explained by the electrode polarization effect, which could reduce the effective potential across the LM.

Conversely, the increased commutation period (0.15 s and 0.20 s) reduces the LM velocity due to intermittent movement. The longer commutation periods could interrupt the consistent generation of the surface tension gradient across the LM due to intermittent pauses for sequential activation of the electrode, reducing the effectiveness of the electrowetting actuation. This leads to a less efficient transfer of momentum to the LM and thus lowers its velocity. Noticeably, LM shows the lowest velocity with a minimal increase across the applied voltage for a 0.2 s commutation period, and an abrupt increase in velocity is observed at 16 V, suggesting a larger voltage requirement to overcome the longer waiting time associated with this commutation period.

In this context, we define the critical voltage of the LM traveling inside a circular microchannel as the minimum voltage for the seamless completion of a full cycle. Figure 3C shows the critical voltage required for LM of different volumes to run continuously at different commutation periods. It can be inferred that smaller droplets demand a higher critical voltage to ensure seamless channel traversal compared to the larger ones. For example, a 5 µL LM droplet requires a voltage of 10 to 14 V, while a 20 µL droplet requires only 4.5 to 7.0 V. This could be explained based on the rate of charge distribution on the LM surface, which is influenced by the droplet size and the temporal characteristics of an applied electric field.

Further, the rate of charge distribution is also dependent on the time constant (τ = RC), a product of resistance (R) and capacitance (C) of the LM-electrolyte system, both of which vary with the droplet size. Thus, smaller droplets exhibit higher resistance due to lower surface area, resulting in a longer time constant. We believe the time constraints for charge redistribution on LM surface cause an insufficient surface gradient to drive the LM forward, necessitating a higher critical voltage for continuous motion. Hence, as the LM volume increases, the critical voltage goes down.

However, as shown in Fig. 3C, the critical voltage increases overall when the LM volume exceeds 15 µL. We hypothesized that this is due to the increase in the viscous drag forces of the larger droplet, thus requiring higher critical voltages to drive the LM seamlessly. These observations highlight the importance of droplet size, temporal factor of the applied field, and charge redistribution dynamics in understanding the critical voltage of LM and its continuous motion in microchannels.

Next, we investigated the effect of electrode polarization on LM velocity by varying electrode sizes. Figure 3D demonstrates LM droplet velocity under varying electrode areas. It can be observed that the velocity of the LM increases as the electrode area increases. Larger electrode surface areas reduce polarization impedance, which decreases resistance and capacitance at the electrode–electrolyte interface. This leads to lower voltage drops and more efficient charge redistribution, producing a stronger surface tension gradient that propels the LM faster. The effect of electrode area on the polarization was verified by measuring the voltage across electrodes of different cross-section areas as shown in Fig.S1 (see Online Resource 7).

Notably, the highest velocity was observed for a fixed electrode area with a 0.1 s commutation period, while the velocity reduced at longer commutation periods due to slower charging and discharging of the EDL around the electrodes [68]. Moreover, surface oxidation of the LM also impacts its movement, with prolonged commutation periods leading to greater oxidation and slower motion. Fuchs et al. previously showed that pulsed signals enhance LM motion by effectively mitigating the accumulation of surface oxide on LM [69].

Our six-electrode system uses a dynamic strategy where the current is sequentially applied with commutation periods ranging from 0.1 s to 0.4 s in 0.05 s increments. Shorter commutation periods enhance LM motion by limiting oxide accumulation, while longer periods slow down the LM through surface oxidation and a viscous drag force, which is described by $F= \frac{1}{2}\rho CA{\upsilon }^{2}$, where $\rho$ is the LM density, $C$ is the drag coefficient, $A$ is cross-sectional area of LM, and $\upsilon$ is displacement velocity. As observed in Fig. 3D the LM moved significantly faster at 0.1 s commutation period compared to 0.35 s and 0.40 s. While shorter commutation periods generally enhance the LM speed, there is a threshold beyond which the LM fails to maintain synchronization with the electric field, disrupting smooth cyclic motion. For instance, at commutation periods shorter than 0.1 s, the LM displayed erratic behavior with intermittent or chaotic motion. This occurred because the electrodes switched faster than the LM could traverse between them—essentially, the next electrode would activate before the LM reached the previous one.

In such cases, the commutation period thus must align closely with the LM’s position to maintain continuous, controlled motion. When this synchronization is disrupted, the LM fails to move consistently, resulting in non-uniform intermittent motion. This highlights the need for a balance between LM velocity and commutation period to ensure that the LM remains in phase with the applied electric field for effective, seamless cyclic motion. More sophisticated methods for real-time synchronization between the LM position and the commutation period could be implemented in future work to improve the optimization further.

The actuation speed is also affected by the volume of LM injected inside the channel. While theoretical models of LM movement within microchannels often treat the LM as a spherical object [45, 51], the actual shape is determined by both the channel dimensions and the electrolyte density. The LM conforms to the channel shape due to its fluidic properties, characterized by a kinematic viscosity in the range of 2.5 × 10⁻⁷–7.5 × 10⁻⁷ m^2^ s⁻^1^ [70, 71]. Wang et al. reported that the critical voltage for LM actuation is inversely proportional to the square of the LM radius, assuming constant parameters such as A_current_, q_0_, ρ, L, η, ρ_diff_ for a given electrolyte concentration [45].

Consequently, the acceleration of LM becomes inversely proportional to the square root of its radius [45, 51], implying that smaller droplet sizes require a higher potential difference to induce a sufficient surface tension differential [52, 72]. We controlled the volume of LM injected inside the channel using a microsyringe pump with nanoliters precision. Figure 3E shows the influence of LM volume on the actuation speed for three channel widths for varying commutation periods. For LMs with volumes less than 5 μL, oscillatory motion was observed, where the LM failed to travel the entire channel length under the applied electric field. The velocity was measured by tracking the displacement of the LM in one stroke of the motion under an applied bias voltage of 8.7 V.

The LM velocity increases with volume till it reaches a maximum at 10 μL. The smaller LM volume has a higher surface area-to-volume ratio, which is affected by resistance forces such as viscous drag and friction along the channel walls, leading to lower velocity. These resistance forces are overcome with larger LM volume, as the larger surface area allows for more efficient charge redistribution and a stronger surface tension gradient, allowing for faster movement. As we gradually increase the volume, the value of A_gap_ (the area between the LM and the channel wall) decreases, resulting in higher pressure [20]. Conversely, larger LM volumes (20–25 μL) experienced greater internal resistance and drag from the channel walls, reducing their velocity relative to smaller droplets (10–15 μL). This behavior indicates that larger LMs require a higher pressure difference to sustain forward motion, further contributing to the observed reduction in velocity for LM volumes in the upper range [58].

Our study indicates that the LM could actuate continuously for about nine hours with our current device parameters. To ensure continuous actuation, we replenished the electrolyte in the channel with the same solution concentration at regular intervals of 30 min. We captured images using a high-speed camera to calculate the velocity of LM before and after refilling the electrolyte, as shown in Fig. 3F. The values of LM velocity after refilling are calculated by averaging the velocity of the droplet for four complete rotations in the channel.

An increase in LM velocity was observed after replenishing the channel with fresh electrolytes. This can be attributed to increased surface ions around the LM after electrochemical reactions with the droplet. The LM actuation continues due to the formation of EDL, which modifies the surface tension of the LM to deform and drive in a direction that minimizes the surface area until the continuous ion depletion in the vicinity of LM weakens the EDL, which is aligned with the previous research [20].

We have successfully demonstrated the ability of our system to run for about 9 h, which is a substantial improvement to the existing work, where the maximum demonstrated CEW in a circular conduit was about 1 h [22]. However, prolonged actuation can lead to LM dissolution upon reacting with the water in the electrolyte solution. This dissolution process leads to the production of gallates on the LM surface such as [Ga(OH)_4_]^−1^,which could lead to the loss of the eutectic ability of the LM [20, 51]. For example, when the gallium content in the device is reduced, the melting point and, hence, the surface tension of the LM are compromised [38, 39]. In other words, these gallates can increase the surface roughness and potentially alter the interfacial interaction essential for indefinite LM actuation. This could potentially explain the deterioration of our device performance after 9 h of continuous actuation.

Additionally, the effect of electrolyte concentration on the velocity of the LM was examined by varying the NaOH concentration from 0.1 M to 0.4 M while maintaining a fixed input voltage and fixed LM volume. The velocity of the liquid metal increases with increasing NaOH concentration for a fixed commutation period, as shown in Fig. S2 (see Online Resource 7). However, a higher concentration could lead to increased gas generation, negatively affecting system performance. Thus, we selected 0.4 M NaOH as the optimal concentration that provides a good balance between maintaining sufficient surface tension force for effective LM motion and minimizing unwanted bubble formation. Besides, the velocity of LM is also affected by the temperature of the electrolyte. The characterization of the pH of the electrolyte as a function of temperature shows an inverse relation, as shown in Fig. S3 (see Online Resource 7).

**Fig. 4:**
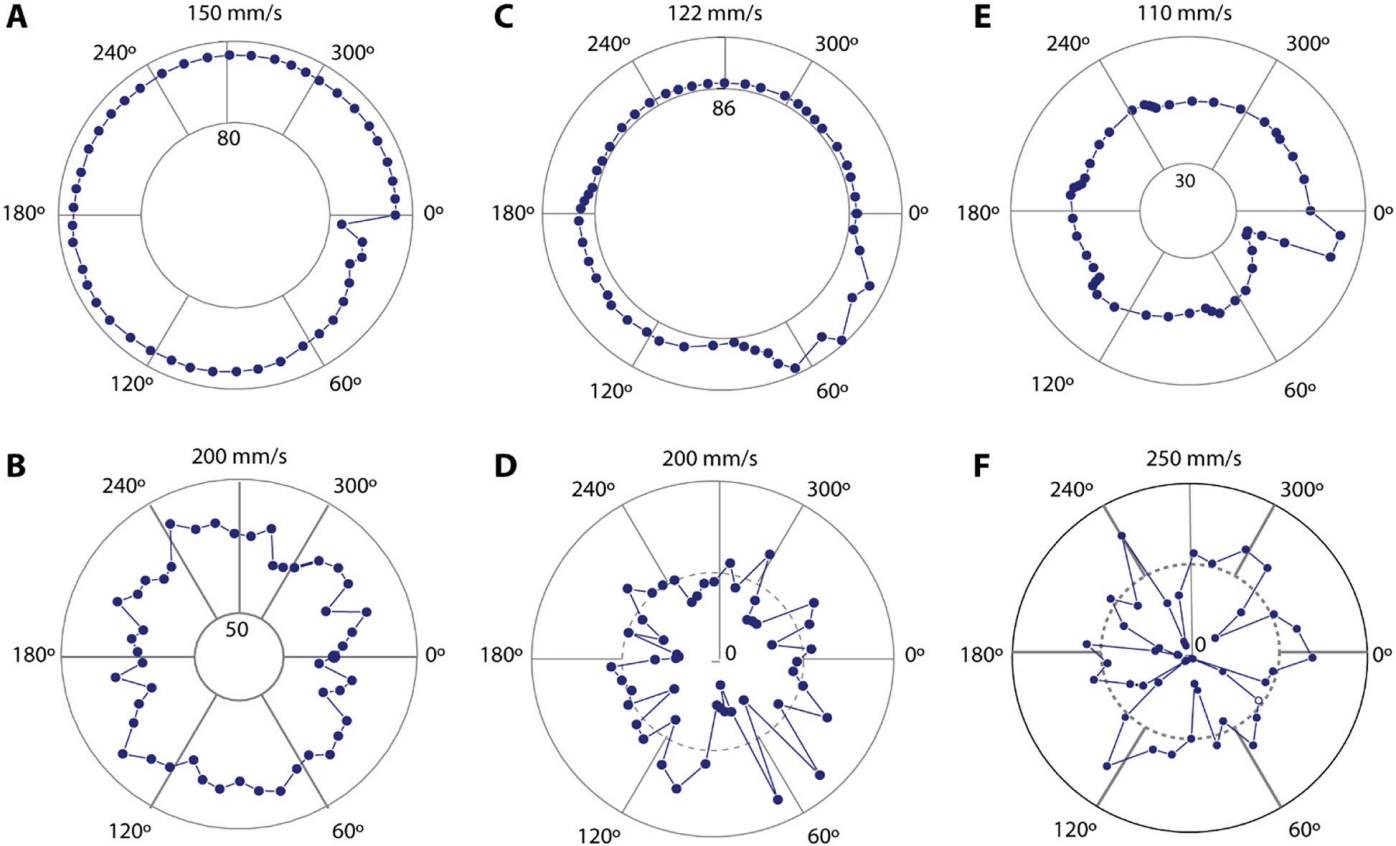
The characterization of the average and instantaneous velocity of the liquid metal. **A** The average velocity of liquid metal when a 0.10 s commutation period is applied on the device. The initial jump of the actuation is due to the excess charges on the surface of the liquid metal before reconfiguration. **B** The instantaneous velocity of liquid metal at a 0.10 s of time-period. **C** Average velocity of the liquid metal at a 0.15 s time which shows a larger deviation on the velocity at different time periods compared to 0.1 s. **D** Corresponding instantaneous velocity of the liquid metal showing a larger delay at the junction of the electrode. **E** Average velocity of the liquid metal at 0.20 s of the of the electrical actuation. **F** Instantaneous velocity of the liquid metal at 0.1 s of electrical actuation. The negative values indicate that the liquid metal moves in the reverse direction at the electrode junctions

We further investigated the motion of the LM through a comprehensive analysis of the average and instantaneous velocity of the droplet within the circular channel. The device was mounted on a flat acrylic plate, and videos were captured on the bottom side of the device. Timestamps were meticulously recorded, such as the time for the LM to transit through a particular electrode and its return to the same electrode after a complete cycle. This allowed us to split the motion of the LM into discrete CEW motions by investigating the position of the LM at equal intervals. For a complete cycle of rotation comprising 48 timestamps, the input voltage was maintained at 8.7 V. The LM volume, channel width, depth, electrolyte concentration, and the area of electrodes were tuned to 10 μL, 3 mm, 6 mm, 0.4 M NaOH, and 27.7 mm^2^, respectively. Figure 4A, C, and E show the average velocity of the LM inside the channel at a commutation period of 0.10 s, 0.15 s, and 0.20 s, while the corresponding instantaneous velocity of LM is shown in Fig. 4B, D, and F.

To enable seamless, smooth motion of the LM, the frequency of commutation of the six electrodes needed to be synchronized. To characterize that, tests were performed at three distinct actuation times—0.10 s, 0.15 s, and 0.20 s, which indicates the duration of time for which each electrode is powered. The time required for the LM to complete a full cycle was divided into 48 equally spaced intervals. Images were captured at those time intervals from the high-speed camera videos, and then, the angular displacement of the LM from the original position was calculated via image processing.

When the commutation period was decreased, the delay in LM movement was notably reduced. Figure 4A and B show the average and instantaneous velocity under 0.10 s of excitation, demonstrating the relatively smooth motion due to accelerated electrochemical dynamics, a stronger surface tension gradient, and effective charge redistribution. Interestingly, at 0.20 s, a negative displacement occurred when the LM reached the electrode junction, as shown in Fig. 4E. This backward movement is linked to charge reconfiguration and the electrocapillary effect; after the electric field is removed, redistributed charges briefly create a surface tension gradient in the opposite direction, driving the LM backward. Moreover, the consistency of LM movement under 0.10 s was confirmed over 30 cycles, demonstrating minimal velocity variation compared to 0.15 and 0.20 s. This suggests that shorter commutation periods reduce the time needed for charge reconfiguration and electrolyte disruption, leading to a relatively smoother, uninterrupted motion in each cycle.

**Fig. 5:**
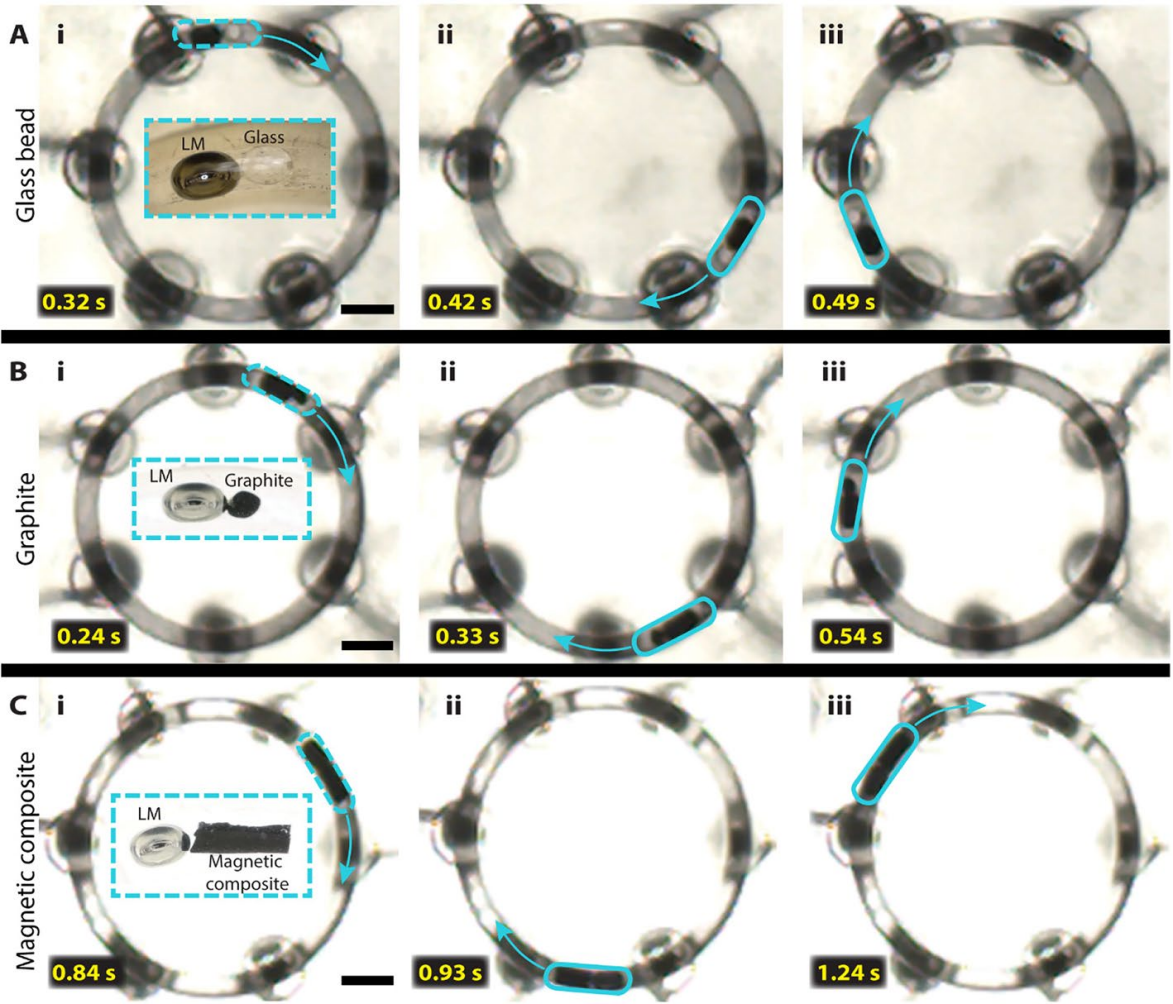
The motion of liquid metal can be utilized to achieve cargo transport of non-conductive, conductive, and magnetic composite materials (**A**) Transport of a glass bead as the non-conductive material (**B**) Transport of graphite chip as a conducting material and (**C**) figure showing the delivery of a magnetic composite. The scale bars are 5 mm. The inset on the figures shows a clearer view of how the LM is aligned with the different types of cargo in front

**Fig. 6:**
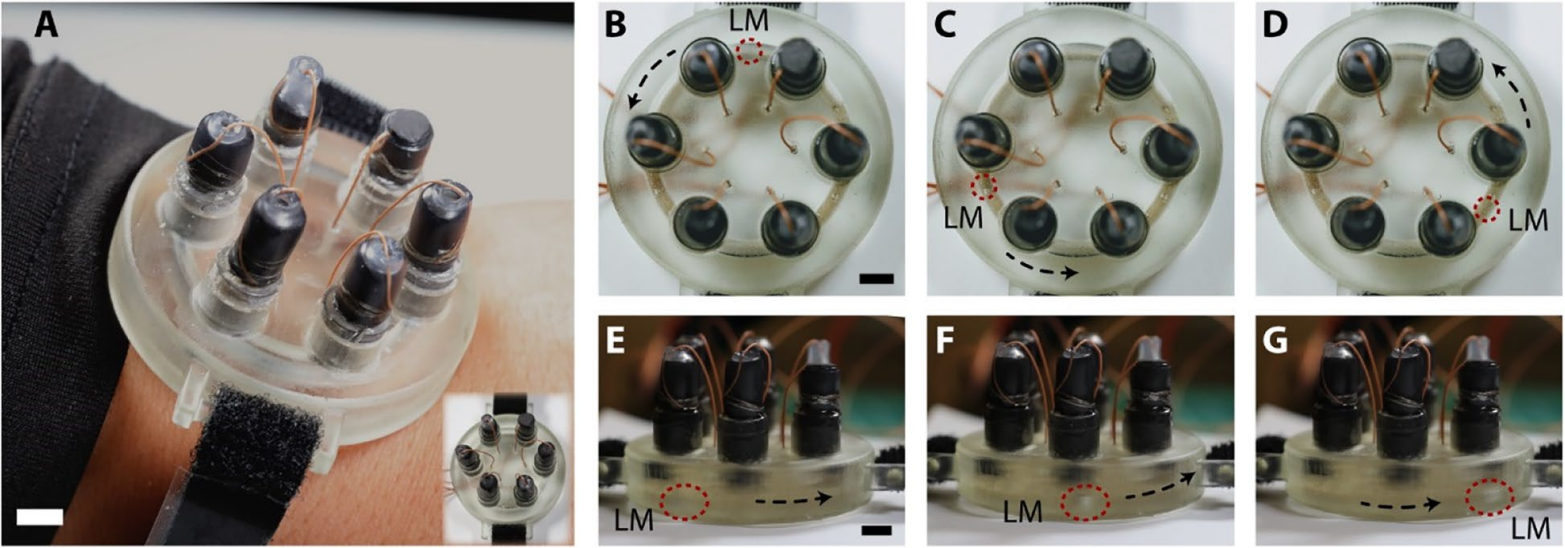
A proof-of-concept demonstration of CEW in the wearable form factor of a watch. **A** The CEW device as worn on the wrist. The inset shows the complete device with the enclosed circular channel and the electrodes. The thin 32 Ga wires are connected to the electronics pack (**B-D**) Time series images of the top view of the device showing continuous actuation of the LM. **E–G** Time series images of the side view of the device showing continuous actuation of the LM. The position of the LM is highlighted in the dashed circle along with the direction of motion which can be tuned as needed. The scale bars are 5 mm

Finally, to explore the potential application of the CEW device, we conducted proof-of-concept experiments with graphite chips (conducting), glass (non-conducting), and a polymer-magnetic composite. It is worth noting that the material used as cargo needs to be denser than the electrolyte to transport in the channel. For example, less dense PDMS is a poor cargo for 0.4 M NaOH solution as it floats on the electrolyte’s surface. Figure 5A (i-iii) shows the time series images of spherical glass beads of 2 mm in diameter being transported inside the microchannel (see Online Resource 2 for video). Figure 5B (i-iii) are the time series images of graphite chip 1.4 mm thick and 2 mm long inside the microchannel (see Online Resource 3 for video), and Fig. 5C (i-iii) are the time series images of transport of magnetic composite from an end of the microchannel to the other (see Online Resource 4 for video). These experiments demonstrated the capability of our CEW platform to carry different types of cargo from one location to another. We believe that our six-electrode design system for gallium-based LM actuation could be a powerful tool for biomedical applications. Additionally, we fabricated an enclosed conduit of similar dimensions and integrated it into a wearable device in the form factor of a smartwatch, as shown in Fig. 6. Figure 6A demonstrates the capability of using our platform as a wearable device targeted for use in biomedical applications. Tests conducted on this wearable platform demonstrated the seamless cyclic motion of the LM (see Online Resource 5), confirming its functionality in a compact and wearable format. Figure 6 (B-D) highlights the time series images of the top view of the device showing LM actuation. Figure 6 (E–G) highlights the time series images of the side view of the device showing LM actuation. Videos of this device in operation are highlighted in the supporting information (see Online Resource 5).

**Fig. 7:**
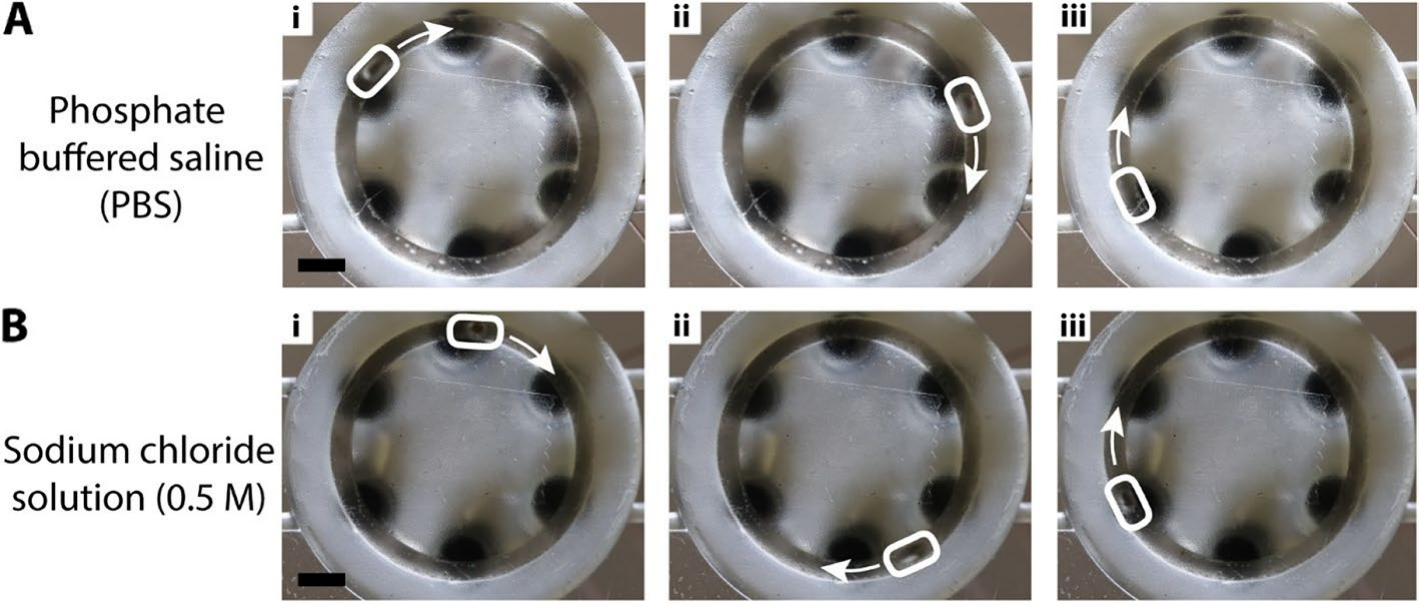
Demonstration of CEW on our proof-of-concept device with alternate biocompatible electrolytes (**A**)(i-iii) Time series images of the bottom view of the device showing continuous actuation of the LM in phosphate buffered saline (**B**)(i-iii) Time series images of the bottom view of the device showing continuous actuation of the LM in 0.5 M aqueous NaCl solution. The scale bars are 5 mm

One critical aspect of wearable devices is the safety and compatibility of the electrolyte. Although NaOH is highly corrosive, its selection was based on the need for a well-characterized electrolyte known to reliably assist in electrowetting phenomena. This choice allowed us to validate our system and provided a reliable benchmark for comparing our device’s behavior and performance with prior research in the CEW domain. To address the biosafety and long-term sustainability concern of the CEW system, we explored biocompatible alternatives for electrolytes, including sodium chloride aqueous solution (0.5 M NaCl, pH 7.8) and phosphate-buffered saline (PBS, pH 7.4) solutions. Actuation tests conducted in both these electrolytes demonstrated successful LM actuation, with the LM drop consistently traversing the circular channel, as shown in the time-series images in Fig. 7 and also highlighted through the video in the supporting information (see Online Resource 6). Further characterization of these electrolytes was out of the scope of this current work.

Another important aspect of long-term actuation is the lifespan of gallium-based liquid metals as they undergo degradation and oxidation in the electrolyte. Earlier research using CEW with Galinstan and NaOH conducted inductively coupled plasma mass spectrometry (ICP-MS) and theoretically estimated a potential lifetime of about 41 days, after which the LM loses its eutectic ability due to gallium loss [20]. They also estimated that the LM can theoretically work for about 100 days in NaCl solution and at least 34 days in PBS buffer. Although we used a different gallium-based LM (eGaIn) with higher gallium content (75.5%) than Galinstan (68.5%), we believe that our system can also achieve a prolonged lifespan until the gallium content in the LM goes below 59.6%, when the eutectic ability is lost [38]. Additionally, other techniques, such as coating the LM with nanoparticles [52, 73], can be leveraged to suppress or mitigate the formation of the oxide layer, allowing for consistent control of surface tension over more extended periods. Future work will focus on improving techniques to extend the lifespan of our CEW device further.

With this work, we have demonstrated prolonged LM actuation through CEW that can serve as a valuable platform for wearable applications, such as microfluidic pumps for transdermal fluid extraction, drug delivery systems, personalized thermoregulation, and health monitoring systems. Future work can focus on advancing the current platform towards real-time wearable applications. For instance, ultra-thin polydimethylsiloxane (PDMS) membranes can be integrated with our cyclic channel and used for peristaltic pumping, enabling fluid extraction and continuous physiological monitoring. Additionally, our system can transport immiscible fluids carrying therapeutic drugs, demonstrating potential in drug delivery while isolating fluids from the electrolyte. The CEW system can also support personalized thermoregulation, leveraging LM’s thermal conductivity to create wearable cooling and heating solutions for precise and efficient temperature control in a wearable device. By leveraging the system’s CEW capabilities, compact design, and extended operational lifespan, these applications could be further developed into practical, user-friendly devices tailored to meet diverse real-world needs.

## Conclusion

In this work, we successfully demonstrated extended duration, low-voltage actuation of LM within a 3D-printed circular channel. By synchronizing the application of a pulsed DC electric field across the electrodes, we were able to actuate the liquid metal at about 150 mm/sec under a voltage of 8.7 V, maintaining this performance for approximately nine hours. This extended operational lifetime is a significant improvement over the previous work, overcoming a key barrier for CEW actuation to be integrated into wearable and soft electronics. In addition to the relatively lower voltage and power requirements, the ability to control the direction and speed of LM actuation by adjusting electrical pulses enables bidirectional flow control. Further, our result suggests a broad range of potential applications, such as in cargo transport, as demonstrated by the ability of the LM to move electrically conductive, insulating, and magnetic composite materials. Future studies can focus on optimizing system parameters, LM compositions, and electrolytes, ultimately enabling the miniaturization of the device architectures and allowing the integration of low-voltage electro-mechanical systems into a wearable form factor.

## Supplementary Information

Supplementary Material 1.

Supplementary Material 2.

Supplementary Material 3.

Supplementary Material 4.

Supplementary Material 5.

Supplementary Material 6.

Supplementary Material 7.

## Data Availability

The code and data that support the findings of this study are available upon reasonable request from the authors.
