# Supplementary material for: The continuous actuation of liquid metal with a 3D-printed electrowetting device
Source: Med X. 2025 Apr 1;3(1):9. doi: 10.1007/s44258-025-00052-8 (PMC11958460; doi:10.1007/s44258-025-00052-8)
Supplement: Supplementary file 7 — Supplementary Material 7. [file 44258_2025_52_MOESM7_ESM.docx]

**The continuous actuation of liquid metal with a 3D-printed electrowetting device**

**Samannoy Ghosh**^†1^**, Rajan Neupane**^†1^ **Dwipak Prasad Sahu**^†1^**, Jian Teng**^1^**, Yong Lin Kong**^1^*

^1^Department of Mechanical Engineering, Rice University, Houston, Texas 77005, USA

^†^equal contributions.

*Corresponding author E-mail: kong@rice.edu

**Effect of electrode surface areas on polarization impedance:**

The relationship between electrode surface area and polarization impedance is well-established in electrochemical theory. Increasing the electrode surface area reduces both the resistance and interfacial capacitance at the electrode-electrolyte interface [1]. A larger electrode area facilitates more charge accumulation, which improves the charge transfer process and reduces polarization effects. Consequently, this results in lower impedance, smaller voltage drops across the electrode, and improved charge redistribution at the interface. Earlier research has shown that reducing the electrode surface area increases the interfacial impedance due to a higher charge density and limited active surface for ion exchange [1]. To validate this theory in the context of our device, we have measured the DC voltage across electrodes with varying surface areas. Specifically, we tested three different electrode surface areas under the fixed input voltage of 8.7 V. As shown in **Figure S1**, we observed that the measured voltage across the electrodes increases as the cross-sectional area of the electrode increases. This observation aligns with the theoretical findings reported in prior research, indicating that larger electrode areas reduce resistance at the electrode-electrolyte interface [1]. Consequently, this reduction leads to a lower polarization impedance and enhanced charge redistribution. These improvements are directly linked to enhanced LM actuation performance.


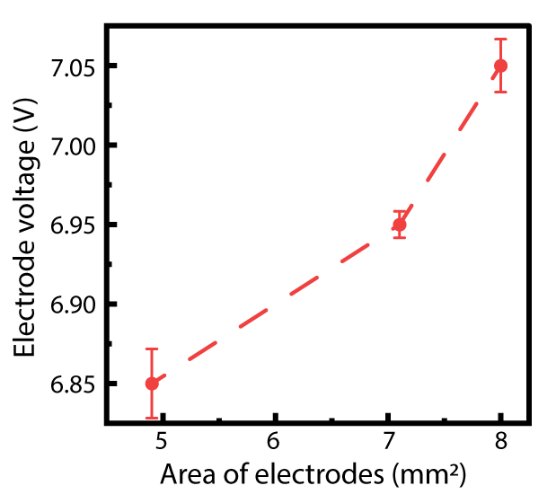


Figure S1. Measured voltage across electrodes for different cross-sectional area of electrodes.

**Effect of electrolyte concentration on the velocity of the liquid metal:**

A preliminary test was conducted to examine the effect of electrolyte concentration on the velocity of the liquid metal before selecting NaOH concentration of 0.4 M. Specifically, we varied the NaOH concentration from 0.1 M to 0.4 M while maintaining a fixed input voltage and fixed LM volume. **Figure S2** shows that the velocity of the liquid metal increases with increasing NaOH concentration for a fixed commutation period. This aligns with the theoretical predictions, as the surface tension force on the LM is proportional to the initial charge per unit area, as shown in Equation 7 in the manuscript.

$$F_{\gamma}=\frac{4\pi r^{2}q_{0}A_{current}V_{electrode}}{\left( A_{current}-\frac{2}{3}\pi r^{2} \right)L},$$

where q_0_ is the initial charge per unit area of the EDL before applying external electric potential. The initial charge is a function of the electrolyte concentration, the type of LM, and the temperature of the electrolyte solution [2,3]. When the concentration of the electrolyte is increased, the ionic conductivity as well as the surface charge density increases. This increases q_0_ and in turn leads to a higher surface tension force, thereby increasing the velocity of the LM. Additionally, we observed that at concentrations lower than 0.4 M, the LM actuation becomes intermittent, showing a tendency to stop and oscillate. This behavior indicates insufficient surface tension force at lower concentrations, which affects the continuous motion of the LM under the applied voltage.

Our objective has been to utilize low NaOH concentrations to minimize hydrogen bubble generation while simultaneously optimizing LM actuation. Higher concentrations could lead to increased gas generation, negatively affecting system performance. Thus, we selected 0.4 M NaOH as optimal concentration that provides a good balance between maintaining sufficient surface tension force for effective LM motion and minimizing unwanted bubble formation.


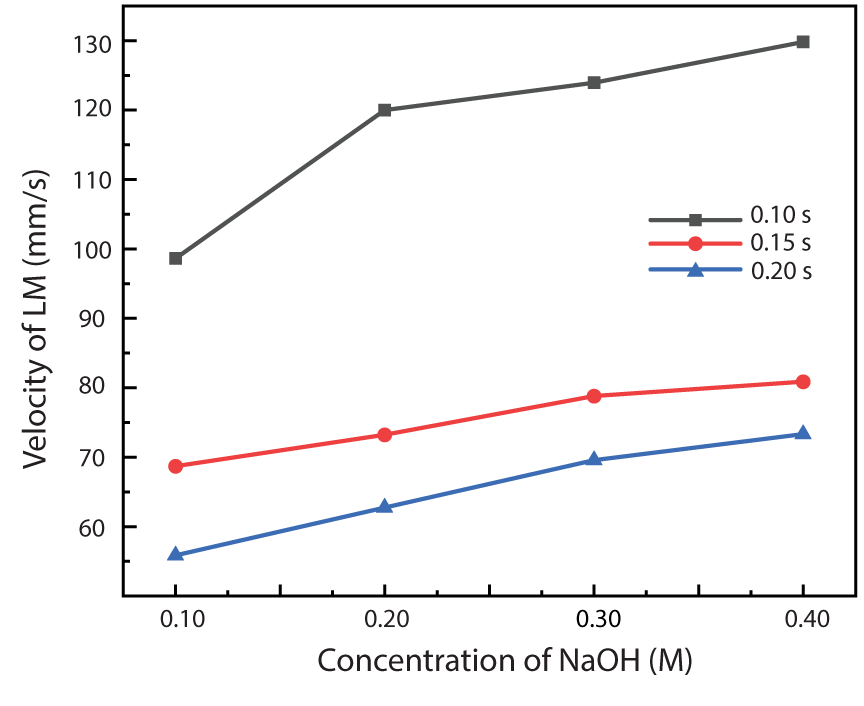


Figure S2. Velocity of LM in circular channel when immersed in different concentrations of sodium hydroxide electrolyte.

**Effect of temperature on the velocity of the liquid metal:**

To study the effect of temperature on the electrolyte, the pH of the electrolyte as a function of temperature was measured between 23 °C (room temperature) and 60 °C, and observed a decrease in the pH of the solution, as seen in **Figure S3**. This can be attributed to the faster dissociation of the H^+^ ions, which decreases the pH. The reduction of pH decreases the ionic strength of the solution, thereby reducing the surface charge density [4]. The reduction in q_0_ directly impacts the surface tension force as described in the Equation 7 in the manuscript,

$$F_{\gamma}=\frac{4\pi r^{2}q_{0}A_{current}V_{electrode}}{\left( A_{current}-\frac{2}{3}\pi r^{2} \right)L}$$

To compensate for the reduced surface charge density at higher temperatures, the device can be optimized by increasing the applied voltage while keeping other parameters constant. The impact of temperature on device performance is anticipated to be minimal, as the device is unlikely to operate above 60°C in wearable applications.


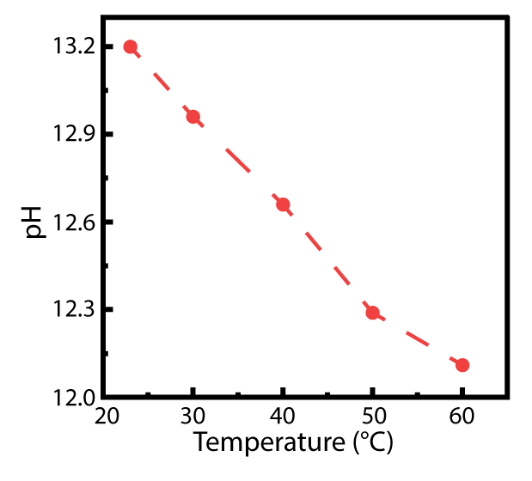


Figure S3. Measured pH of electrolyte (0.4 M NaOH) as a function of temperature

**Effect of biocompatible electrolytes on liquid metal actuation:**

We explored biocompatible alternatives for electrolyte including sodium chloride aqueous solution (0.5 M NaCl, pH 7.8) and phosphate-buffered saline (PBS, pH 7.4) solutions. Actuation tests conducted in both these electrolytes demonstrated successful LM actuation, with the LM drop consistently traversing the circular channel (**see Online Resource 6**). **Figure 7** in the manuscript illustrates the LM’s movement within the circular channel using these electrolytes, underscoring the feasibility of our system using biocompatible electrolytes.

However, the required voltage for actuation was significantly higher (> 21 V) compared to NaOH electrolyte. This observation is aligned with the theory for electrowetting phenomena, which is governed by the interaction between the charge density and the electric double layer (EDL) at the LM-electrolyte interface. In electrolytes with higher bulk ion concentration (such as NaOH, which has a higher pH), the abundance of hydroxide ions (OH⁻) significantly enhances the charge density (𝜌) within the EDL, leading to strong electrostatic interaction at the interface. A higher charge density directly increases the charge per unit area on the LM surface (q_0_), which, according to Equation 7 in the manuscript, leads to an increase in the surface tension force,

$$F_{\gamma}=\frac{4\pi r^{2}q_{0}A_{current}V_{electrode}}{\left( A_{current}-\frac{2}{3}\pi r^{2} \right)L}\text{.}$$

This facilitates charge redistribution on the LM surface, enabling effective modulation of surface tension at relatively lower applied voltages, as shown in our current prototype. Conversely, in near-neutral electrolytes like PBS, the ionic strength and bulk ion concentration are comparably lower than in NaOH. This leads to reduced q_0_ and a decrease in the net surface tension force. Therefore, the electric field must compensate by applying a higher voltage to generate a surface tension gradient for LM actuation, as observed in our case where we had to apply higher voltage for LM actuation. These results demonstrate the feasibility and flexibility of our CEW system in utilizing biocompatible electrolytes through minor optimizations.

**References**

1. Ahmed R and Reifsnider K 2011 Study of influence of electrode geometry on impedance spectroscopy *Int. J. Electrochem. Sci.* **6** 1159–74
2. Brown M A, Goel A and Abbas Z 2016 Effect of electrolyte concentration on the stern layer thickness at a charged interface *Angew. Chem. Int. Ed.* **55** 3790–4
3. Handschuh-Wang S, Chen Y, Zhu L, Gan T and Zhou X 2019 Electric actuation of liquid metal droplets in acidified aqueous electrolyte *Langmuir* **35** 372–81
4. Shao Y, Helstrom M, Yllo A, Mindemark J, Hermansson K, Behler J and Zhang C 2020 Temperature effects on the ionic conductivity in concentrated alkaline electrolyte solutions *PCCP* **22** 10426-10430
